# Enhancing a SARS-CoV-2 nucleocapsid antigen test sensitivity with cost efficient strategy through a cotton intermembrane insertion

**DOI:** 10.1038/s41598-023-31641-5

**Published:** 2023-03-22

**Authors:** Diego Rinaldi Pavesi Nicollete, Rafael Benedetti, Beatriz Arruda Valença, Keyla Kaori Kuniyoshi, Thainá Caroline Schuartz de Jesus, Ava Gevaerd, Erika Bergamo Santiago, Bernardo Montesanti Machado de Almeida, Sérgio Renato Rogal Júnior, Marcus Vinícius Mazega Figueredo

**Affiliations:** Research and Development Department, Hilab, Hilab Campus, José A. Possebom, 800, Curitiba, Paraná 81270-185 Brazil

**Keywords:** Infectious diseases, Diagnostic markers, Diagnosis

## Abstract

Lateral flow antigen tests have been widely used in the Covid-19 pandemic, allowing faster diagnostic test results and preventing further viral spread through isolation of infected individuals. Accomplishment of this screening must be performed with tests that show satisfactory sensitivity in order to successfully detect the target protein and avoid false negatives. The aim of this study was to create a lateral flow test that could detect SARS-CoV-2 nucleocapsid protein in low concentrations that were comparable to the limits of detection claimed by existing tests from the market. To do so, several adjustments were necessary during research and development of the prototypes until they were consistent with these criteria. The proposed alternatives of increasing the test line antibody concentration and addition of an intermembrane between the conjugate pad and the nitrocellulose membrane were able to increase the sensitivity four-fold and generate a new rapid test prototype called “lateral flow intermembrane immunoassay test” (LFIIT). This prototype showed an adequate limit of detection (2.0 ng mL^−1^) while maintaining affordability and simplicity in manufacturing processes.

## Introduction

In December 2019, several patients were hospitalized in Wuhan City, Hubei province, China, with pneumonia-like respiratory symptoms of unknown etiology, with subsequent studies showing compelling evidence that Wuhan’s Huanan seafood and wildlife market could be related to the outbreak. The causative agent was identified as a betacoronavirus of sarbecovirus subgenus, belonging to Orthocoronavirinae subfamily, and was initially named as 2019-nCoV, which later would be renamed as SARS-CoV-2^1^.

Coronaviruses are enveloped single-stranded positive sense RNA viruses that affect mainly the respiratory tract, but are also capable of causing neurological, enteric and hepatic effects^2,3^. Up until December 2019, six coronaviruses were known to infect humans, with four of them causing mild flu-like symptoms, while the other two were responsible for highly pathogenic epidemics: SARS-CoV and MERS-CoV^4–6^. Likewise, in March 2020, the World Health Organization (WHO) declared the novel coronavirus disease (COVID-19) a global pandemic, caused by SARS-CoV-2^7,8^.

SARS-CoV-2 shares 79.5% genetic similarity with SARS-CoV and viral particle assembly is dependent on four main structural proteins: membrane (M), nucleocapsid (N), envelope (E) and spike (S) proteins, in addition to nonstructural and accessory proteins^9,10^. SARS-CoV-2 main method of infection relies on Spike glycoprotein, which shows a trimeric structure on the surface of the virus. It is comprised of two subunits: S1 and S2, being that S1 has a receptor binding domain (RBD) and is responsible for binding with angiotensin-converting enzyme 2 (ACE2), the main receptor for SARS-CoV-2 in host cells^11^. For this reason, Spike protein has been targeted with great attention for research and development of therapeutic drugs and vaccines for COVID-19^12^. However, Spike protein is also the structural protein that is most affected by genetic mutations on different variants of SARS-CoV-2, which leads to important changes in antibody recognition, affecting diagnostic tests and vaccines^13^. Similar studies showed that N protein is much more conserved between variants and its mutations do not affect antibody binding in rapid diagnostic tests so far, making it a better target for SARS-CoV-2 antigen rapid testing^14^.

Rapid testing offers several advantages over traditional laboratory methods that require trained personnel, equipment and take longer time for obtaining results^15^. Moreover, antigen testing tends to correlate well with the contagiousness status of the patient, revealing itself as a powerful tool for isolating the infected and reducing viral spread, when compared to molecular methods^16^.

**Figure 1:**
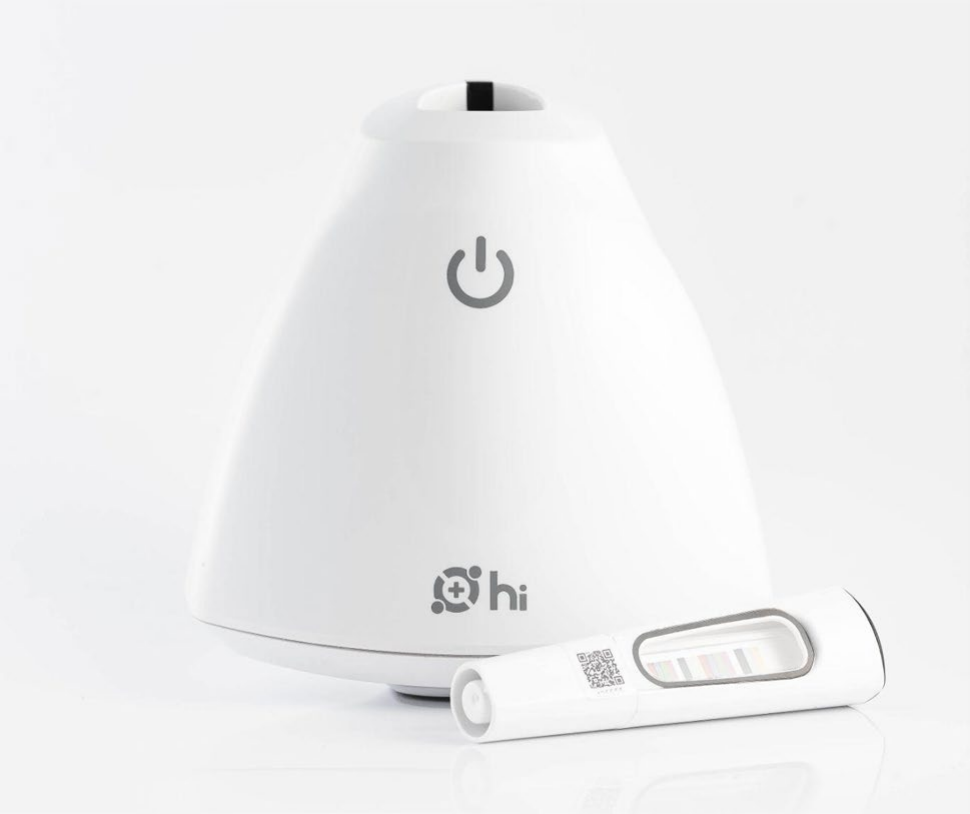
Hilab Flow, a device capable of performing lateral flow and vertical flow point of care tests.

In this scenario, Hilab is a remote clinical laboratory that is able to perform several clinical tests in different human samples with the aid of portable proprietary equipment developed by the company’s team of multidisciplinary researchers. Hilab service uses internet of things (IoT) and artificial intelligence (AI) technologies to provide double verified results that are collected from the samples through the devices and analyzed by deep learning networks that provide a first insight into a patient's health status. This data is then verified by health professionals that validate, sign and release the reports back to the patient’s e-mails and cellular phones in minutes. Hilab Flow (Fig. 1) is one of the company’s main devices, a small handheld analyzer (12.4 × 12.4 × 12.7 cm; 0.45 kg) operating in a multi-methodology scenario that is able to perform immunochromatography, immunofluorescence, colorimetry and dry chemistry point of care tests with the company’s service technology as a background. Gasparin et al. recently published a work describing usage of Hilab Flow for hemoglobin measurement by vertical flow dry chemistry colorimetry in whole blood as part of a complete blood count test developed by Hilab researchers, although most of the tests performed in Hilab Flow are based on lateral flow immunoassays (LFIAs)^17^.

The device is patented and registered in Brazilian Health Regulatory Agency (ANVISA—registration number 80583710007), and is equipped with CMOS sensors and multi spectral light sources capable of illuminating the capsule containing the strip in the interior of the device. Upon light emission and signal reception, the data is captured and processed through a proprietary software that employs image processing, deep learning, neural networks and other AI techniques to calculate signal-to-noise ratios via measurement of peak-forming optical density of the bands.

LFIA are well recognized for their good performance for point of care testing, eliminating the need for highly trained professionals or laboratory infrastructure, and providing affordability with their low cost^18^. These attributes make LFIAs especially effective in reaching populations with low resources and/or lack of structure, as well as high demand testing scenarios such as health emergencies or pandemics^19^. "Sandwich” type LFIAs are paper-based diagnostic devices that function with immunochromatographic principles for the detection of clinically relevant analytes in a variety of samples. After dispensing the sample with a running buffer on the sample pad, the analyte, if present, interacts with dried reagents such as nanoparticles conjugated with antibodies and migrates by capillary action until the immune complexes reach the test line, comprised of a second antibody for the same analyte, which will bind the reagents and show a reactive signal. The test strip also contains a second line with specific antibodies for a control conjugate, to demonstrate that the strip is functioning correctly, acting as an internal control^20^.

In order to guarantee good management of the viral spread in COVID-19, rapid tests used for screening must show good sensitivity for detection of SARS-CoV-2 N protein, avoiding false negatives and providing correct information about infection status. With this in mind, the concept of limit of detection as the lowest concentration of analyte that can be safely differentiated from blank is an important tool for the evaluation of sensitivity in diagnostic tests^21^, and this information may lead to the identification of the need for sensitivity amplification strategies when developing these solutions. Clinical sensitivity performance of several SARS-CoV-2 N antigen diagnostic tests commercially available in Brazil were compared in a recent paper published by Freire et al., in which the authors described the sensitivity variation in a range from 9.8 to 81.1%. Low sensitivities from these screening tests could hinder mass test efficacy in controlling SARS-CoV-2 viral spread by allowing infected individuals to stay in close contact with healthy counterparts^22^.

In this work we described a comparative information about sensitivity-boosting strategies applied in the development of a gold nanoparticle LFIA for the detection of SARS-CoV-2 antigen (N protein). Firstly, an increase in antibody concentration in the test line was able to double the sensitivity of the prototype. To further enhance the sensitivity and reach or surpass comparable limits of detection to benchmarking tests of the market, a second strategy was necessary. In this regard, two routes were evaluated: increase of concentration of colloidal gold conjugate and insertion of a cotton intermembrane in order to increase antibody-antigen interaction time.

## Materials and methods

### Test results readout and interpretation

All test prototypes described in this work were developed and tested by the team of research and development in laboratory innovation (immunochromatography) at Hilab, Brazil, and evaluated in Hilab Flow (ANVISA registration no. 80583710007) as well as naked eye for best correlation and applicability.

### Reagents and equipment

Colloidal gold nanoparticles (40 nm) were purchased from Arista Biologicals and used as a detection label for both test and control antibodies. The conjugates were analyzed for absorbance with a Nanodrop UV–Vis Spectrophotometer (Thermo Scientific). Phosphate buffered saline (PBS) used for dilution of the antibodies and preparation of buffers was purchased from Sigma Aldrich (CAT P7059). Capture antibodies for test and control lines were purchased from Arista Biologicals (COVID-19 nucleoprotein mAb and goat anti mouse IgG, respectively). For lamination of the LFIIT sheets, Standard 14 glass fiber from Cytiva (22 mm × 50 m) was used as both sample and conjugate pads (355 µm thickness at 53 kPA). For the intermembrane strategy, Whatman MF1 bound glass fiber filter and CF3 cotton linter material (322 µm thickness at 53 kPA) were purchased from Cytiva (22 mm × 50 m). The nitrocellulose membrane CN140 was purchased from Sartorius (25 mm × 100 m, thickness 225-255 µm, capillary speed of 95–155 s/40 mm). CF5 cotton linter material (954 µm thickness at 53 kPA) was used as a wick pad and purchased from Cytiva (27 mm × 50 m). Level of detection testing was performed with Fapon SARS-CoV-2 recombinant N protein FPZ0513, expressed in *E. coli*. Test line intensity comparison was performed with Zeptometrix SARS-CoV-2 antigen control 0810590CFHI. Dispensing of test/control lines and CGC was performed with a lateral flow reagent dispenser supplied by JH.Bio (Model: XYZ3020). Drying of all materials was performed with a forced air oven incubator purchased from Solidsteel (model SSB.O.DU-342L). Cutting of the sheets into lateral flow test strips was accomplished in an automatic guillotine cutter (Xangai Jiening Biotec, Model CM3030). To centrifuge the CGC up to 30 OD, a microcentrifuge model NT805 purchased from Novatécnica was utilized.

### Preparation of AuNP conjugates

Colloidal gold nanoparticles (40 nm) were passively conjugated to commercially available SARS-CoV-2 anti-nucleocapsid antibodies (Arista Biologicals, Allentown, PA). The stock colloidal gold conjugate (CGC) concentration was 20 OD with batch-dependent pH of 8.5–9.2. When applicable, the colloidal gold was concentrated to 30 OD in a microcentrifuge (8000×*g*, 8 °C for 20 min). To concentrate the CGC, after centrifugation, the proportional volume of supernatant was discarded without resuspending the pellet. After pipetting, the pellet was resuspended in an ultrasonic bath and the new OD was confirmed with a Nanodrop One UV–Vis Spectrophotometer, at 530 nm. A mix of 80% anti-N CGC and 20% control CGC (mouse IgG) was sprayed (10 μL cm^−1^) on the conjugate pads for assembly onto the test strips.

### Fabrication of lateral flow intermembrane immunoassay test

The LFIIT has modifications when compared to a conventional lateral flow, for intermembrane insertion. In this way, the model layout was based on a sample pad, conjugate pad, intermembrane, nitrocellulose membrane, and absorbent pad. To prepare the sample pad, a pre-treatment was performed with a sodium tetraborate solution (50 mM, pH 7.5, 1% BSA), with immersion in the buffer solution for about two hours (~ 25 °C, ~ 50% RH). To dry the sample pad, the material was placed in a forced air-drying oven incubator (~ 20% RH, 37 °C) for 2 h. The conjugate pad was sprayed with the CGC blend in a 20 OD concentration with sucrose (5.0%) and trehalose (5.0%), with a lateral flow reagent dispenser. After impregnation, the CGCs were also dried in a forced air drying oven incubator (~ 20% RH, 37 °C) for two hours. Capture antibodies were dispensed as test and control lines in the nitrocellulose membrane using the same lateral flow reagent dispenser. The antibodies were diluted in a PBS 0.01 M (pH 7.4) solution to the desired concentration and the impregnated sheets were dried in a forced air-drying oven incubator (~ 20% RH, 37 °C) for two hours. After drying the components, the sample pad (1.5 cm × 0.4 cm), conjugate pad (1.0 cm × 0.4 cm), intermembrane (0.7 cm × 0.4 cm), nitrocellulose (2.5 cm × 0.4 cm) and absorbent pad (1.5 cm × 0.4 cm) were sequentially laminated over 0.2 mm thickness standard adhesive backing cards and the sheets were cut in 4.0 mm lateral flow strips with an automatic guillotine cutter.

### Simulated testing procedure and analytical performance

To simulate the test procedure, and to obtain the corresponding analytical responses, the commercial control/calibrator samples, described below, were diluted in the extraction buffer provided in a dropper bottle to the concentrations or proportions described. To start the test, four drops of the extraction solution (~ 80 μl) are dispensed directly in the indicated region of the sample pad. Results were read after 15 min with a visual (naked eye) interpretation as “reactive” with an apparent test line and “nonreactive” for no apparent test line, and a Hilab Flow analysis resulting as “positive” or “negative”.

In order to perform the sensitivity and limit of detection (LOD) experiments, a purified SARS-CoV-2 nucleocapsid protein (FPZ0513, Fapon, Taiwan, CN) was used. The protein was diluted in 80 μl of the prototype running buffer in pre-defined concentrations: 10 ng mL^−1^, 5 ng mL^−1^, 3.75 ng mL^−1^, 2 ng mL^−1^ and 1 ng mL^−1^, corresponding to a protein inoculum of 0.8 ng, 0.4 ng, 0.28 ng, 0.16 ng and 0.08 ng per test, respectively. Testing was performed initially using five replicates, and for better understanding of cutoffs and limits, the replicates were increased to 20 in critical threshold concentrations (near the cutoff). The concentration defined as the limit of detection of the test was considered when 95% of replicates (19/20) showed reactive test lines, regardless of the intensity obtained^23^. Regarding the commercial antigen control material, Zeptometrix SARS-Related Coronavirus 2 (SARS-CoV-2) Isolate: Hong Kong/VM20001061/2020 Culture Fluid (Heat Inactivated) (catalog number 0810590CFHI) was used and the applicable proportional dilutions were made in the assay extraction buffer.

### Ethics declaration

This study was performed with the rapid test prototype only, and no human samples were used for its optimization whatsoever. Only a commercial purified recombinant SARS-CoV-2 N protein and a control material made of inactivated viral particles from culture fluid were used, therefore no ethics committee clearance or patient consent is applicable.

### Statistical analysis

Statistical data were analyzed and plotted using the Graphpad Prism software (version 7, San Diego, CA, USA). A one-way ANOVA test was carried out to compare the effects of different sensitivity enhancement strategies on the intensity of the test lines captured by measurement of optical density with the Hilab Flow device. When appropriate, a post-hoc Tukey test was performed. The significance level was set at p ≤ 0.05.

## Results and discussion

The original prototype of the test described in this work was developed following a step-by-step optimization of individual components, with the best candidates in each experiment advancing to the next development stage, until the full dipstick strip and running buffer were defined, with a conjugate concentration of 20 OD sprayed in a dispense rate of 10 μL cm^−1^ and a test line of 1.5 mg mL^−1^ antibody striping. After the development of all the components of the test kit, it is necessary to assess the preliminary sensitivity of the prototype to verify its compatibility with the intended use and the concentration of the analyte to be detected. This verification usually results in further optimization necessities for the test components until requirements are met^24^. The strategies implemented by the group to address this analytical sensitivity issue are described below.

### Strategy 1: sensitivity enhancement through increase of test line antibody concentration

The first prototype was challenged with a LoD test recommended in the FDA template for SARS-CoV-2 antigen test development, which establishes the LoD as the lowest concentration of protein detected by 19 of 20 test replicates in the experiment^23^. Screening of the initial LoD in this non-optimized prototype resulted in 7.5 ng mL^−1^ of N protein, which was short in terms of sensitivity compared to data from literature and similar rapid tests from the market^25,26^.

After these experiments, an adjustment in the test line antibody concentration was implemented, doubling the amount to 3.0 mg mL^−1^, which was an acceptable impregnation rate regarding cost (the increase was still within target pricing for the future product, when compared to local competitors) and effective antibody binding to nitrocellulose without displacement during strip manipulation and test execution, as higher concentrations tend to negatively affect strength of interactions between protein and nitrocellulose and could possibly lead to false positives^27^. Initial screening for the LoD, was performed with the protocol of quintuplicate of tests for four initial concentrations of N protein: 100.0, 10.0, 5.0, and 1.0 ng mL^−1^, with visual and equipment interpretation of results, as shown in Table 1.

**Table 1:** Limit of detection screening for 3.0 mg mL^−1^ test line LFIA prototype 1 (P1 + 3.0).

Prototype	Replicate	N protein concentration (ng mL^−1^)	Visual result	Hilab flow result
P1 + 3.0	1	100.0	Reactive “+++”	Positive
	2		Reactive “+++”	Positive
	3		Reactive “+++”	Positive
	4		Reactive “+++”	Positive
	5		Reactive “+++”	Positive
	1	10.0	Reactive “++”	Positive
	2		Reactive “++”	Positive
	3		Reactive “++”	Positive
	4		Reactive “++”	Positive
	5		Reactive “++”	Positive
	1	5.0	Reactive “+”	Positive
	2		Reactive “+”	Positive
	3		Reactive “+”	Positive
	4		Reactive “+”	Positive
	5		Reactive “+”	Positive
	1	1.0	Weak reactive	Positive
	2		Weak reactive	Positive
	3		Weak reactive	Positive
	4		Nonreactive	Negative
	5		Nonreactive	Negative

P1 + 3.0: prototype 1, with 3.0 mg mL^−1^ test line. Non-reactive: no apparent test line. Reactive semiquantitative analysis is defined as “+++” for a very strong signal, “++” for a strong signal, “+” for moderate signal and “Weak reactive” for a faint line.

The results showed that LoD of the prototype was between concentrations of 5.0 and 1.0 ng mL^−1^ of N protein. Since the concentration of the antibody of the test line was doubled, an intermediate limit of detection was hypothesized as being half of the initially established limit (7.5 ng mL^−1^).

**Figure 2:**
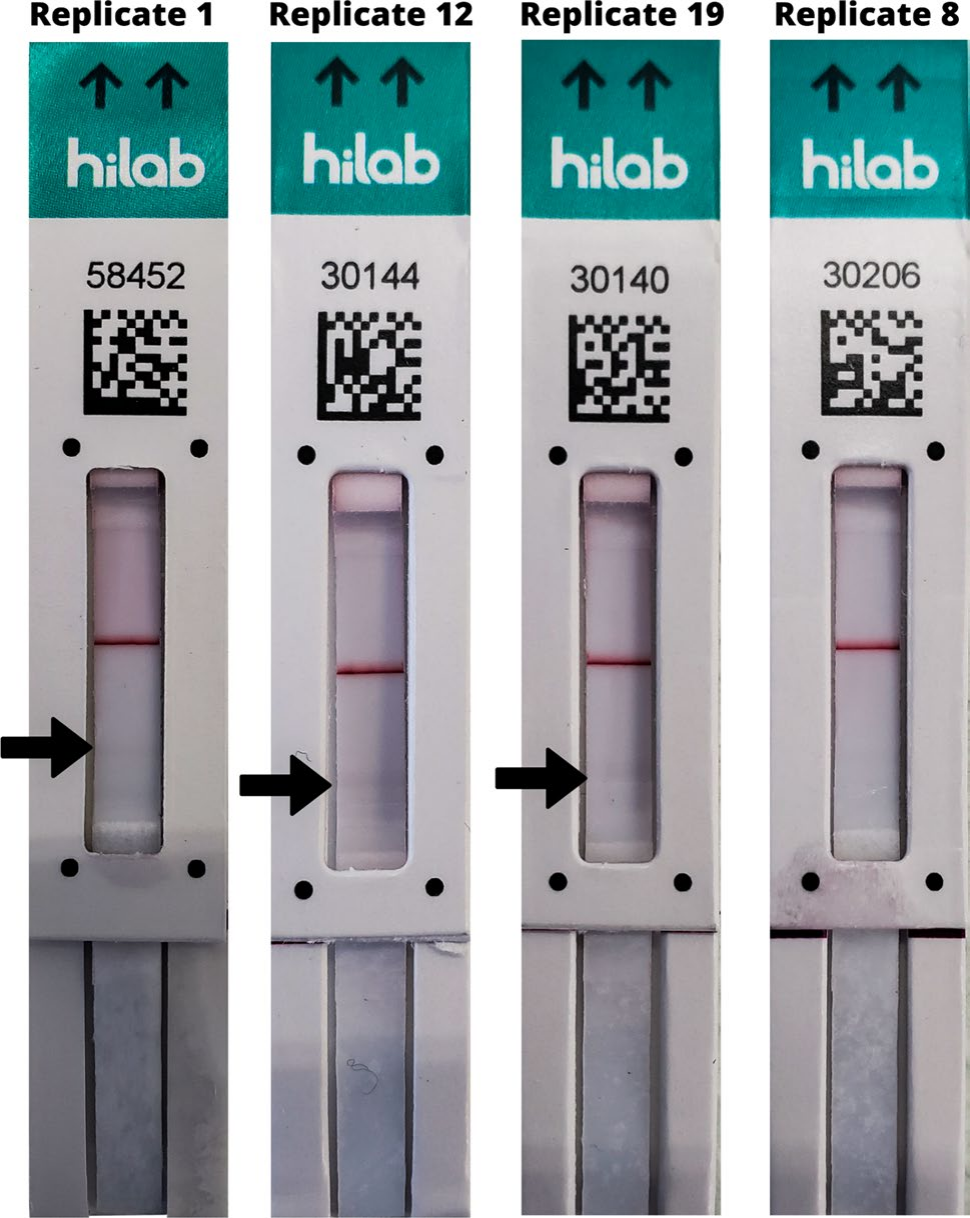
P1 + 3.0 Limit of detection results for 3.75 ng mL^−1^ of SARS-CoV-2 N protein. Replicates 1, 12 and 19: faint lines as reactive signals are indicated by the arrows. Replicate 8: no apparent test line (non-reactive). Control line shows the proper function of the test strips.

Therefore, to verify the true limit of detection between 5.0 and 1.0 ng mL^−1^, an experiment with 20 replicates using a 3.75 ng mL^−1^ concentration of N protein was performed. The results are shown below in Table 2, and a representative image of the strips used to perform these studies is shown in Fig. 2.

**Table 2:** Limit of detection definition for 3.0 mg mL^−1^ test line LFIA prototype 1 (P1 + 3.0).

Prototype	Replicate	N Protein concentration (ng mL^−1^)	Visual result	Hilab flow result
P1 + 3.0	1	3.75	Reactive	Positive
	2		Reactive	Positive
	3		Reactive	Positive
	4		Reactive	Positive
	5		Reactive	Positive
	6		Reactive	Positive
	7		Reactive	Positive
	8		Nonreactive	Negative
	9		Reactive	Positive
	10		Reactive	Positive
	11		Reactive	Positive
	12		Reactive	Positive
	13		Reactive	Positive
	14		Reactive	Positive
	15		Reactive	Positive
	16		Reactive	Positive
	17		Reactive	Positive
	18		Reactive	Positive
	19		Reactive	Positive
	20		Reactive	Positive

P1 + 3.0: Prototype 1, with 3.0 mg mL^−1^ test line. Results defined qualitatively as Reactive for any signal shown on the test line and Nonreactive for no apparent test line.

Therefore, the strategy to increase the capture antibody concentration resulted in a proportional increase in sensitivity, establishing good competitiveness of the prototype among other commercially available tests with the 3.75 ng mL^−1^ LoD^22,25,26^. This prototype was moved on to the next stage of development and optimization.

### Strategy 2: further sensitivity enhancement through increase of CGC concentration versus intermembrane insertion

Following the achievement of the first sensitivity enhancement to match competitor tests from the market, a second sensitivity optimization was still possible and viable to guarantee a reliable diagnosis of early and low viral load infections. Increasing the conjugate OD could address this issue, by increasing the amount of gold-labeled antibody per strip. However, this strategy involves an important increase in reagent costs, considering that CGC is one of the costliest components in a lateral flow test^28^. Research on current literature revealed affordable and effective strategies for sensitivity increase in LFIAs by allowing longer reaction time between the analyte and the CGC with the insertion of wettable/porous materials between the conjugate pad and the nitrocellulose membrane, such as sponges^29^, polyester, cellulose and glass fibers^30^, while others apply a similar concept by using a wax barrier to increase antigen–antibody interaction time^31^. The insertion of an extra layer of material is capable of retaining or slowing down the liquid flow through the strip, emulating an incubation step on the fly as antibodies and antigens stay in close contact for a longer time. These geometrical and paper architecture modifications allow manipulation of the LFIA attributes and have an important impact on test sensitivity and overall performance^32^**.**

**Figure 3:**
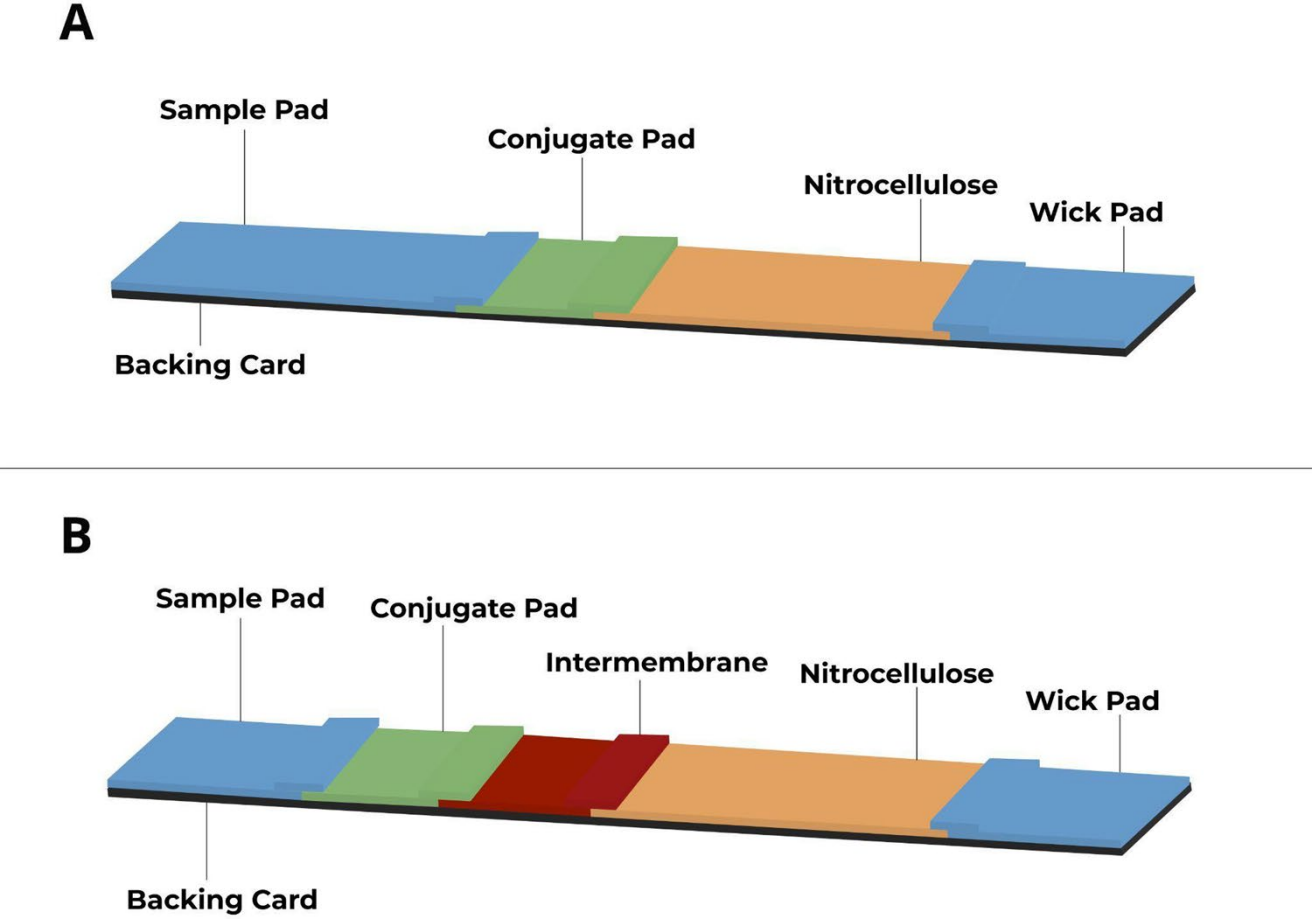
Traditional LFIA and a new proposed design called LFIIT. (**A**) Lateral view of a traditional lateral flow strip, showing the test components and assembly order. (**B**) Lateral view of a LFIIT prototype (insertion of an intermembrane), showing the test components and assembly order. Images not in scale and proportion.

With this in mind, new strip prototypes were generated as described in the materials and methods section, either by concentrating the CGC to 30 OD or by reorganizing the layout of the LFIAs to include an intermembrane (7 mm length) between the conjugate pad and nitrocellulose (Fig. 3B), as opposed to a traditional lateral flow strip arrangement (Fig. 3A).

To evaluate the effect of different materials in the intermembrane composition, a glass fiber (MF1, Whatman) and a cotton fiber (CF3, Whatman) were tested. The new prototypes, named as P2 (original P1 + 3.0), P2 + 30 OD (original with 30 OD CGC), P2 + MF1 (original with MF1 intermembrane) and P2 + CF3 (original with CF3 intermembrane), were first tested using a commercially available antigen control based in an inactivated culture fluid of SARS-CoV-2 (Zeptometrix, NY, EUA), with serial dilutions to measure the initial performance of the prototypes. The results are shown in Table 3.

**Table 3:** Signals obtained for Zeptometrix SARS-CoV-2 antigen control comparing different LFIA prototypes.

Prototype model	Control dilution	Visual result	Hilab flow result
P2	Pure	Reactive “+++”	Positive
P2 + 30 OD		Reactive “+++”	Positive
P2 + MF1		Reactive “+”	Positive
P2 + CF3		Reactive “+++”	Positive
P2	1:16	Weak Reactive	Positive
P2 + 30 OD		Reactive “++”	Positive
P2 + MF1		Weak Reactive	Positive
P2 + CF3		Reactive “++”	Positive
P2	1:64	Weak Reactive	Positive
P2 + 30 OD		Weak Reactive	Positive
P2 + MF1		Weak Reactive	Positive
P2 + CF3		Weak Reactive	Positive
P2	1:128	Nonreactive	Negative
P2 + 30 OD		Nonreactive	Negative
P2 + MF1		Nonreactive	Negative
P2 + CF3		Nonreactive	Negative
P2	Negative	Nonreactive	Negative
P2 + 30 OD		Nonreactive	Negative
P2 + MF1		Nonreactive	Negative
P2 + CF3		Nonreactive	Negative

P2: original P1 + 3.0 (3.0 mg mL^−1^ antibody concentration on test line); P2 + 30 OD: original with 30 OD CGC; P2 + MF1: original with MF1; P2 + CF3: original with CF3 intermembrane; Non Reactive: no apparent test line. Reactive semiquantitative analysis is defined as “+++” for a very strong signal, “++” for a strong signal, “+” for moderate signal and “Weak reactive” for a faint line.

**Figure 4:**
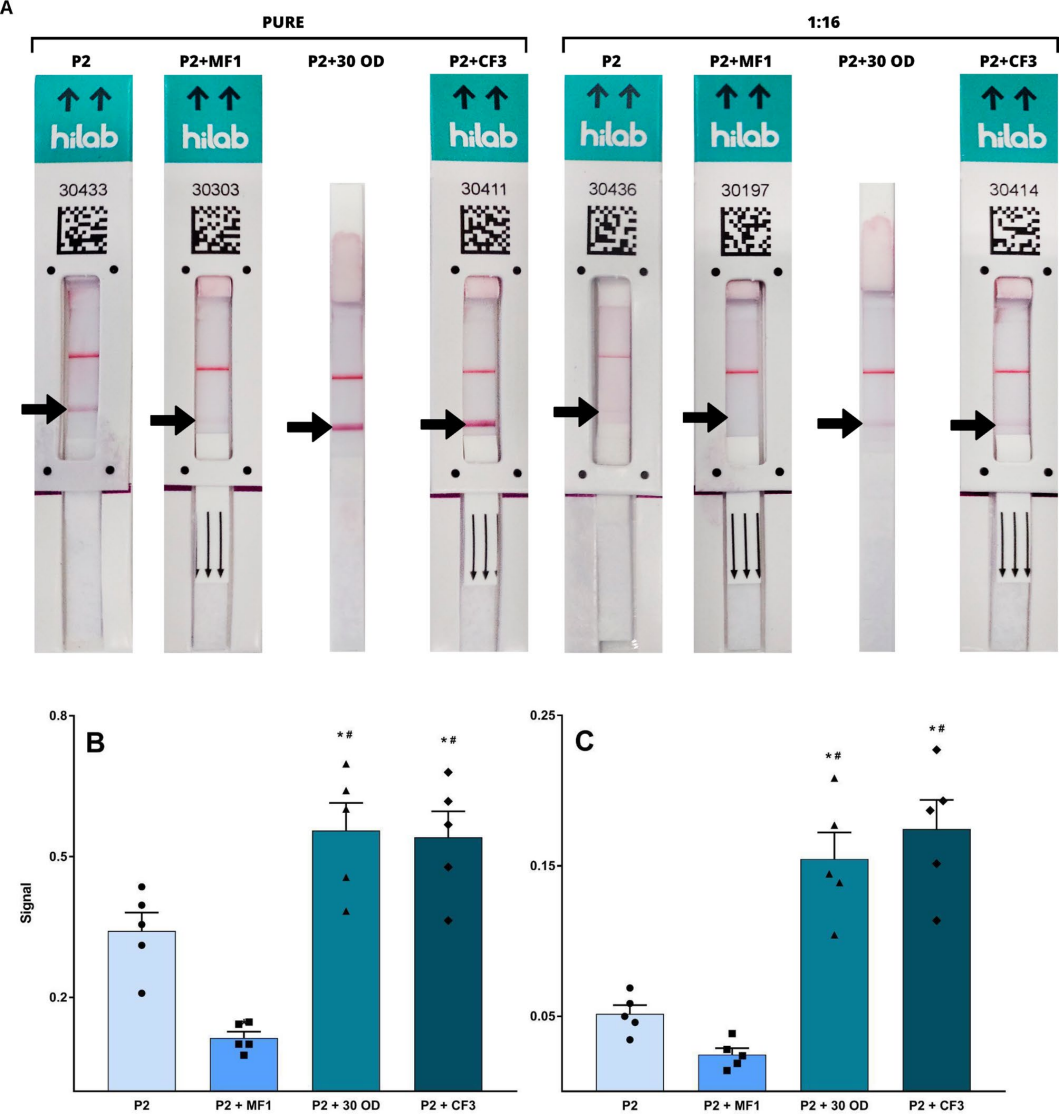
LFIA prototypes signal comparison for a commercially available SARS-CoV-2 antigen control. (**A**) Representative image of the tests. P2: original P1 + 3.0 (3.0 mg mL^−1^ antibody concentration on test line); P2 + 30 OD: original with 30 OD CGC; P2 + MF1: original with MF1 intermembrane; P2 + CF3: original with CF3 intermembrane; Control line shows the proper function of the test strips. (**B**) Bar plot showing statistical comparison between intensities of the test lines on different prototypes using a commercially available SARS-CoV-2 antigen control. (**C**) Signal comparison for 1:16 dilution of the same control material. Signal: Relative intensity units measured by Hilab Flow. # represents a significant difference compared to the P2 group. *Represents a significant difference compared to the P2 + MF1 group. Comparison between P2 + 30OD and P2 + CF3 showed no significant statistical difference. One-way ANOVA and Tukey post hoc test were carried out when appropriate for p < 0.05.

Results with the antigen control revealed that prototypes P2 + 30 OD and P2 + CF3 had similar sensitivity enhancement when compared to P2 prototype. Representative images of the experiment can be observed on Fig. 4A. Comparison of the relative intensities of the test lines captured by Hilab Flow by optical density of the lines (Supplementary Table S1) revealed that both P2 + 30 OD and P2 + CF3 signals were significantly higher than the other prototypes, and also not significantly different from each other (Fig. 4B,C). This result reveals that insertion of the cotton intermembrane was able to potentially increase the signal in the same magnitude of concentration of the CGC to 30 OD, meaning that the longer reaction time between antibodies and antigen provided by the material could result in the same analytical sensitivity or LoD of a 50% CGC increase with a simpler and more reasonable solution.

Despite the promising results shown above using the commercial control, an experiment with SARS-CoV-2 N protein was still necessary to confirm the improvement in the LoD of the prototypes, in addition to the comparison between the candidates for finer decision making about development of the assay.

#### Limit of detection screening between P2 + 30 OD and LFIIT (P2 + CF3)

**Figure 5:**
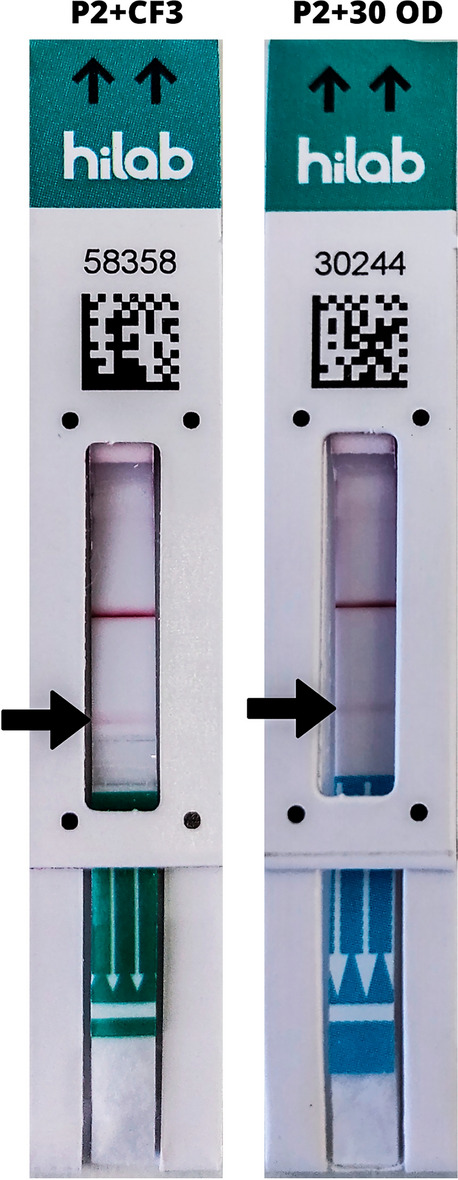
Prototype P2 + CF3 and P2 + 30 OD comparison in Limit of detection using 2.0 ng mL^−1^ protein dilution. P2 + CF3: original prototype with a CF3 intermembrane addition. P2 + 30 OD: original with 30 OD CGC. Faint lines as reactive signals are indicated by the arrows. Control line shows the proper function of the test strips.

To verify the enhanced sensitivity of the new combinations, both P2 + 30 OD and P2 + CF3 prototypes were tested, since they presented the best potential in the previous experiment. SARS-CoV-2 N protein was diluted to a 2.0 ng mL^−1^ concentration, generating a raw amount of 0.16 ng of protein per test after calculation of the dilution in running/extraction buffer, representing half of the concentration established for the last LoD experiment. The results of this LoD screening are shown in Table 4 and visual comparison may be observed in Fig. 5.

**Table 4:** Limit of detection comparison between P2 + 30OD and P2 + CF3.

Prototype	Replicate	N protein concentration (ng mL^−1^)	Visual result	Hilab flow result
P2 + 30 OD	1	2.0	Reactive	Positive
	2		Reactive	Positive
	3		Reactive	Positive
	4		Reactive	Positive
	5		Reactive	Positive
P2 + CF3	1		Reactive	Positive
	2		Reactive	Positive
	3		Reactive	Positive
	4		Reactive	Positive
	5		Reactive	Positive

P2 + CF3: original prototype with a CF3 intermembrane addition. P2 + 30 OD: original with 30 OD CGC. Results defined qualitatively as Reactive for any signal shown on the test line and Nonreactive for no apparent test line.

As observed in Fig. 5, the intensity of the signal on the test line was relatively low for 2.0 ng mL^−1^ concentration of N protein, indicating that the LoD of the prototypes was in proximity of this concentration. Since this experiment showed similar results for both prototypes, the most affordable candidate between them was selected for full LoD testing (P2 + CF3, or LFIIT), by increasing the number of replicates to twenty.

#### Final limit of detection after LFIIT establishment

Standard limit of detection recommended testing protocol was followed for this experiment, as usual. N protein was diluted in the same concentration tested in LOD P2 + 30 OD and P2 + CF3 comparison (2.0 ng mL^−1^). Expected limit of detection was to be defined for at least 19 reactive results in 20 replicates tested (95% detection rate). The results are shown in Table 5.

**Table 5:** Final limit of detection for the LFIIT prototype.

Prototype	Replicate	N protein concentration (ng mL^−1^)	Visual result	Hilab flow result
LFIIT	1	2.0	Reactive	Positive
	2		Reactive	Positive
	3		Reactive	Positive
	4		Reactive	Positive
	5		Reactive	Positive
	6		Reactive	Positive
	7		Nonreactive	Negative
	8		Reactive	Positive
	9		Reactive	Positive
	10		Reactive	Positive
	11		Reactive	Positive
	12		Reactive	Positive
	13		Reactive	Positive
	14		Reactive	Positive
	15		Reactive	Positive
	16		Reactive	Positive
	17		Reactive	Positive
	18		Reactive	Positive
	19		Reactive	Positive
	20		Reactive	Positive

*LFIIT* lateral flow intermembrane immunoassay test (P2 + CF3). Results defined qualitatively as reactive for any signal shown on the test line and nonreactive for no apparent test line.

**Figure 6:**
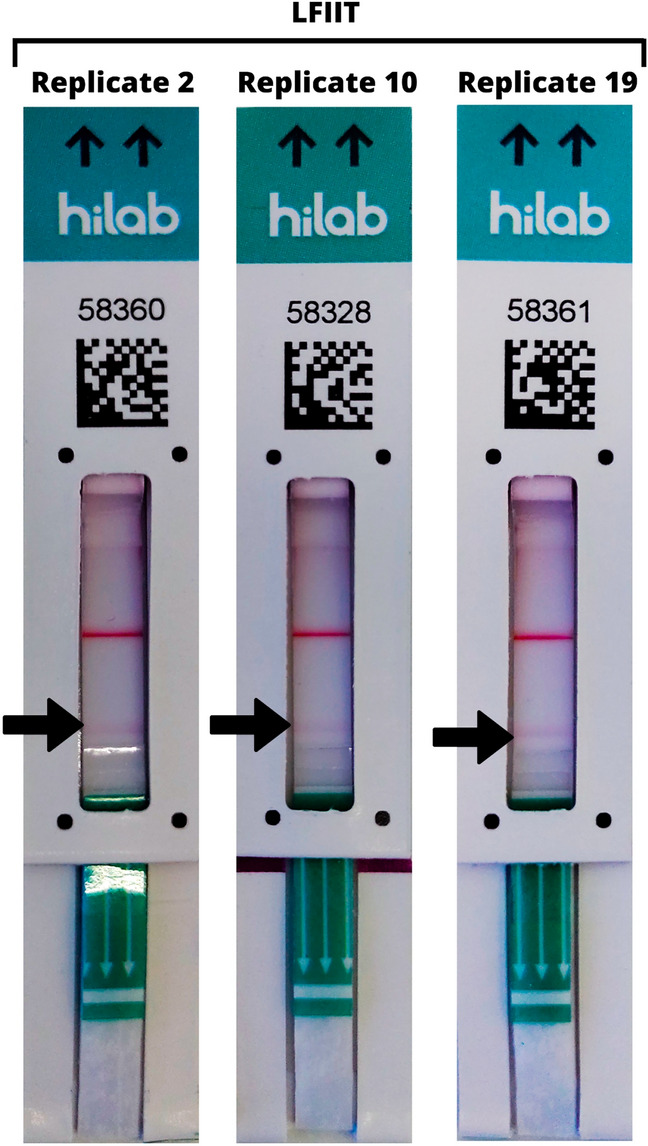
Final limit of detection results for LFIIT prototype with 2.0 ng mL^−1^ of SARS-CoV-2 N protein. *LFIIT* lateral flow intermembrane immunoassay test (P2 + CF3). Faint lines as reactive signals are indicated by the arrows. Control line shows the proper function of the test strips.

Results showed that the LFIIT provided a reactive signal in 19 of the 20 replicates tested, defining the limit of detection of the test as 2.0 ng mL^−1^ of N protein, or 0.16 ng total inoculum of protein per test, thus confirming the increased sensitivity through the use of the intermembrane and ruling out the need for increased colloidal gold. A representative image of the results can be observed in Fig. 6.

### Test cost and sensitivity balance analysis on prototype development decision making

SARS-CoV-2 antigen tests represent an important tool for pandemic management and control, being used extensively for mass screening providing several benefits over laboratory-centered methodologies, especially where turnaround time is critical and immediate results are needed, despite being less sensitive that molecular testing in general^33^. This leads to a cost-effectiveness balance shift in favor of the usage of LFIAs in Covid-19, with estimates suggesting significant reductions in number of cases, hospitalizations, ICU admissions and deaths^34^. However, in order to fulfill this role, the tests must show adequate sensitivity and low cost. These parameters enable mass antigen screening strategies that may be implemented even in asymptomatic individuals, representing surveillance testing for prevention and control of recurring outbreaks^35^.

In our work, surprisingly, the inexpensive and somewhat simple strategy of the intermembrane addition produced similar results to the increase of concentration of the conjugate, providing a second two-fold sensitivity gain to the test. In total, a four-fold sensitivity increase was obtained for the prototype of the Lateral flow intermembrane immunoassay test (LFIIT) while maintaining affordability and simple manufacturing conditions, making it a competitive and viable solution for SARS-CoV-2 antigen testing.

Preliminary cost analysis of the strategies revealed that conjugate concentration would imply in an individual cost of about US$ 7.20 per uncut sheet, while CF3 utilization as an intermembrane in the same quantity of tests would cost US$ 4.61, a 35% cost reduction for the same sensitivity increase and test performance. Furthermore, intermembrane insertion is a much simpler process regarding manufacturing, implying parallel cost reduction with personnel, equipment and infrastructure, since CGC concentration is a critical and time-consuming process.

A third prototype that could potentially increase sensitivity even further was also tested (P2 + CF3 + 30OD), but the limit of detection was the same as the LFIIT prototype (data not shown), possibly due to test line antibody saturation.

Despite being easy to use, scalable and inexpensive, LFIAs tend to have lower sensitivity than traditional immunoassays such as ELISA, due to the dynamic nature of reactions and the kinetics of antigen–antibody interactions. The need to facilitate testing for the public in general leads to LFIAs usually having procedures with few and simple operational steps, without incubation or washing steps, and this could potentially lead to limitations on antigen antibody interactions^36^.

The establishment of the lowest possible limit of detection is also extremely important when developing a SARS-CoV-2 antigen test, since isolation of the patients is highly dependent on antigen test positivity, when transmissibility is much more probable. Antigen tests with optimized limits of detection also tend to be more reliable in guaranteeing that viral load is below an infectiousness threshold when taking the decision to end the isolation period^37^. A comparative analysis between the LFIIT developed in this work and other SARS-CoV-2 antigen tests is shown below, in Table 6.

**Table 6:** Comparison between published SARS-CoV-2 antigen tests limits of detection and LFIIT prototype developed by Hilab.

Test	Manufacturer	Source	Limit of detection (ng mL^−1^ of N protein)
SARS-CoV-2 rapid antigen test (self-test)	Roche, Mannheim, Germany	^25^	6.0
CLINITEST rapid COVID-19 antigen self-test	Siemens Healthineers, Erlangen, Germany	^25^	6.0
Rapid SARS-CoV-2 antigen test card	Xiamen Boson Biotech Co., Xiamen, China	^25^	0.6
Panbio COVID-19 Ag rapid test device (NASAL)	Abbott, Jena, Germany	^25^	6.0
Panbio COVID-19 Ag rapid test	Abbott, Jena, Germany	^26^	Between 5.0 and 10
BIOCREDIT COVID-19 Ag	RapiGEN, St Ingbert, Germany	^26^	250.0
Coronavirus Ag rapid test cassette	Swab; Healgen, Houston, TX, USA	^26^	2.5 or better
COVID-19 Ag RespiStrip	Coris BioConcept, Gembloux, Belgium	^26^	25.0
RIDA QUICK SARS-CoV-2 antigen	R-Biopharm, Darmstadt, Germany	^26^	2.5 or better
NADAL COVID-19 Ag test	nal von minden, Moers, Germany	^26^	Between 5.0 and 10.0
SARS-CoV rapid antigen test	Roche-SD Biosensor, St Ingbert, Germany	^26^	5.0
LFIIT prototype	Hilab, Curitiba, Brazil	***	2.0

***This work.

With these results, it is possible to obtain insights on cost aware sensitivity enhancement strategies that may compensate for the limit of detection challenges found when developing point of care LFIA diagnostic solutions. Antibody concentration increase on the test line was able to initially boost sensitivity to an acceptable level, with the insertion of the intermembrane as a complimentary enhancement to test performance.

## Conclusions

In this work we described strategies that are viable to implement for enhancement of sensitivity and limit of detection of LFIAs, while taking in consideration the costs involved in the process. The 2.0 ng mL^−1^ LoD established for the test prototype in this work is comparable and even superior to most rapid tests for detection of SARS-CoV-2 antigen found on the market, making it an efficient and affordable solution for COVID-19 viral spread containment and decision making in patients’ isolation. Initial sensitivity boost to an acceptable value was achieved with an increase in capture antibody concentration on the test line. Subsequently, insertion of a piece of cotton linter material as an intermembrane for flow rate reduction and increase in antigen–antibody contact time in the prototype doubled the sensitivity of the test, producing the same result as a 50% CGC concentration increase with a much more affordable approach. Total sensitivity enhancement of the test was approximately fourfold compared to the first prototype, starting with 7.5 ng mL^−1^ (0.6 ng of N protein per test) to 2.0 ng mL^−1^ (0.16 ng of N protein per test), with no less than 35% estimated cost reduction on reagents for the final prototype.

## Supplementary Information

Supplementary Information.

## Data Availability

The datasets used and/or analysed during the current study are available from the corresponding author on reasonable request.
