# Supplementary material for: Enhancing a SARS-CoV-2 nucleocapsid antigen test sensitivity with cost efficient strategy through a cotton intermembrane insertion
Source: Sci Rep. 2023 Mar 22;13:4690. doi: 10.1038/s41598-023-31641-5 (PMC10031715; doi:10.1038/s41598-023-31641-5)
Supplement: Supplementary file 1 — Supplementary Information. [file 41598_2023_31641_MOESM1_ESM.pdf]

Supplementary Table S1: Raw data from comparison of the relative intensities of the test lines captured by Hilab Flow for Zeptometrix 0810590CFHI SARS-CoV-2 Antigen control.

Replicate	P2 Pure	P2+ MF1 Pure	P2+ 30 OD Pure	P2 + CF3 Pure
1	0,3966	0,1429	0,6013	0,5681
2	0,3110	0,1003	0,4558	0,6177
3	0,4354	0,1474	0,6974	0,6795
4	0,2090	0,0767	0,3836	0,3637
5	0,3553	0,1004	0,6406	0,4777

Replicate	P2 1:16	P2+MF1 1:16	P2+ 30 OD 1:16	P2+ CF3 1:16
1	0,0499	0,0385	0,1446	0,1868
2	0,0459	0,0186	0,1388	0,1515
3	0,0688	0,0279	0,2083	0,2272
4	0,0344	0,0139	0,1041	0,1136
5	0,0585	0,0237	0,1770	0,1932

	P2 Pure	P2+ MF1 Pure	P2+ 30 OD Pure	P2 + CF3 Pure
Mean	0,3414	0,1135	0,5557	0,5413
CV	25,59%	26,86%	23,63%	22,85%
SD	0,0874	0,0305	0,1313	0,1237

	P2 1:16	P2+MF1 1:16	P2+ 30 OD 1:16	P2+ CF3 1:16
Mean	0,0515	0,0245	0,1546	0,1745
CV	25,23%	38,39%	25,63%	24,85%
SD	0,0130	0,0094	0,0396	0,0434
